# Mechanistic insights into the use of rhubarb in diabetic kidney disease treatment using network pharmacology

**DOI:** 10.1097/MD.0000000000028465

**Published:** 2022-01-07

**Authors:** Yingyuan Gao, Zheng Nan

**Affiliations:** a Internal medicine of traditional Chinese Medicine, Changchun University of Chinese Medicine, Changchun, China; b Internal medicine of traditional Chinese Medicine, Traditional Chinese Medicine Hospital of Jilin Province, Changchun, China.

**Keywords:** diabetic kidney disease, network pharmacology, rhubarb

## Abstract

In this study, we predicted the core active compounds of rhubarb used in the treatment of diabetic kidney disease (DKD) and the related core gene targets and pathways using network pharmacological approaches.

The Traditional Chinese Medicine Systems Pharmacology Database and Analysis Platform was used to identify active compounds of rhubarb. PharmMapper was used to predict the gene targets of active compounds, which were subsequently provided a standard nomenclature using the UniProt database. In addition, DKD-related target genes were predicted using GeneCards, Online Mendelian Inheritance in Man, and Therapeutic Target Database. The genes that were targeted both by rhubarb active compounds and implicated in DKD (hereafter referred to as overlapping target genes) were identified using Venny 2.1. A drug–target–disease network diagram was obtained using Cytoscape and a protein–protein interaction network diagram was constructed using the STRING database. Gene Ontology and Kyoto Encyclopedia of Genes and Genomes enrichment analyses of overlapping target proteins were performed using the Database for Annotation, Visualization and Integrated Discovery Bioinformatics Resources 6.8.

Eighteen core active compounds of rhubarb were extracted, and 136 target genes of rhubarb were identified. Some of the active compounds revealed by the network pharmacological analysis were catechin, aloe-emodin, rhein, and emodin; certain core target proteins suggested by the protein–protein interaction network analysis were AKT1, PIK3R1, and SRC. The overlapping target genes were primarily involved in apoptosis and proteolysis, with the PI3K/Akt signaling pathway identified as significantly enriched.

Network pharmacological strategies were used to identify core active compounds of rhubarb and their related pathways. We believe that our study will provide potential and effective novel targets to identify active compounds of rhubarb for treating DKD.

## 1 Introduction

Diabetes is a chronic, metabolic hyperglycemic disorder and occurs due to impaired secretion or function of insulin, or both. The global prevalence of diabetes was about 8.8% in 2017, with about 7.3% of individuals having impaired glucose tolerance. The highest prevalence of diabetes is reported in China, India, and the United States.^[1]^ Diabetic kidney disease (DKD) or diabetic nephropathy is one of the main complications associated with diabetes. According to a study, 30% to 40% of individuals with diabetes tend to develop DKD despite good control of blood pressure and blood sugar.^[1]^ The China Kidney Disease Network Annual Data Report mentioned DKD as the top-most risk factor for chronic kidney diseases in urban residents in China in 2016.

The Traditional Chinese medicine (TCM) has been used for more than 2,000 years; moreover, it is currently being widely used in clinical practice to treat several diseases including diabetes.^[2–3]^ Rhubarb, a widely used component of TCM, has several medicinal properties. For example, rhubarb extracted from the rhizomes of *Rheum officinale* Baill, *Rheum palmatum* L., and *Rheum tanguticum* Maxim. ex Balf. is known for its medicinal benefits. The primary active components of rhubarb include aloe-emodin, chrysaron, emodin, chrysophanol, rhaponiticin, and D-catechin, which have the main effects of cooling and detoxification, in addition to removing stagnation, improving blood circulation, and preventing blood stasis. Clinically, rhubarb has been used to treat diabetes, DKD, and other metabolic syndromes.^[4–6]^ For example, emodin, a major active component of rhubarb, efficiently prevents oxidative damage to the kidneys of mice with DKD.^[7]^ However, the molecular mechanism contributing to the beneficial effects of rhubarb in treating DKD remains unclear.

Several drugs function by regulating the expression of multiple genes and proteins. For example, mulberry leaves alleviate diabetes by activating the p38 MAPK/NF-κB pathway and inducing autophagy^[8–9]^; *Ginkgo biloba L.* leaves extract prevents brain damage after ischemic stroke by regulating the expression of Bax/Bcl-2 and Caspase-3.^[10]^ Network pharmacology, a pharmacological branch to study the interactions of “drug–target–disease,” was first proposed in 2007 by Andrew L. Hopkins, a pharmacologist from Dundee University, UK.^[11]^ Therefore, the concept of network pharmacology deviates from “one drug, one gene, one disease” principle used in earlier studies. Thus, this concept can be applied to predict the underlying mechanism of decoction pieces of TCM involving multiple targets and pathways.^[12]^

**Figure 1:**
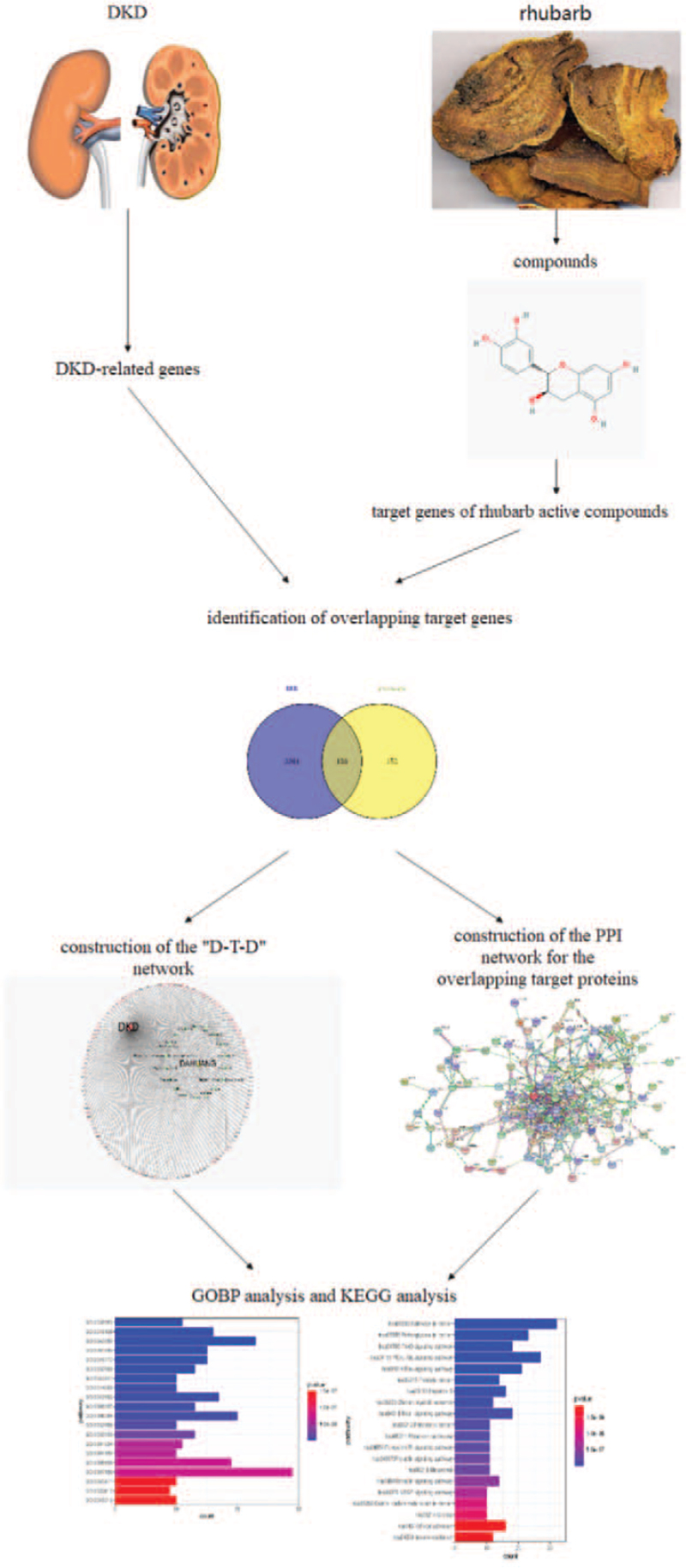
The flowchart of our network pharmacological analysis.

We used network pharmacological approaches to identify the active components of rhubarb. In addition, we predicted the related target genes and key pathways to decipher the underlying mechanism of rhubarb in treating DKD and provide a scientific rationale for further in-depth studies on the therapeutic effects of rhubarb on DKD. Figure 1 shows the flow chart describing our network pharmacological approach.

## 2 Materials and methods

### 2.1 Construction of a library of rhubarb active compounds

The Traditional Chinese Medicine Systems Pharmacology Database and Analysis Platform (TCMSP) provides information on active compounds, targets, and drug–target networks.^[13]^ It contains details of 499 herbal medicines, included in the 2010 edition of Chinese Pharmacopoeia, and their active compounds (more than 29,000). The active compounds were screened for several pharmacological properties including oral bioavailability (OB), drug-like (DL) properties, intestinal epithelial permeability, blood–brain barrier permeability, and half-life.^[13]^

### 2.2 Screening for rhubarb active compounds

We selected OB and DL as the primary parameters to screen active compounds of rhubarb using the TCMSP database. Bioavailability refers to the rate and extent to which a drug is absorbed into circulation. Oral bioavailability reflects the amount of a given drug that enters the circulation, that is, the percentage of oral drug absorbed by the gastrointestinal tract and reaching the blood circulation via the liver.^[14]^ DL reflects how similar a compound is to the known drugs, that is, DL compounds have the potential to behave like drugs. These compounds are known as drug-like molecules or drug analogs. We used “rhubarb” as the keyword to search the TCMSP database using the screening criteria of DL ≥ 0.18 and OB > 30%.^[15]^

### 2.3 Construction of a target gene library for rhubarb active compounds

To predict the active compounds of rhubarb, their two-dimensional (2D) structures were retrieved from PubChem (https://pubchem.ncbi.nlm.nih.gov/) and submitted to the PharmMapper service platform. We selected only “human protein targets (v2010,2241)” for “select target set.” The results were organized according to the fit value, and the top 50 targets were selected. Finally, the names of the retrieved target genes were replaced with their corresponding official symbols given in the UniProt database (https://www.uniprot.org/).^[16]^

### 2.4 Construction of a library for DKD-related genes

We used the “diabetic kidney disease” as the keyword to search the GeneCards (https://www.genecards.org/),^[17]^ Online Mendelian Inheritance in Man (OMIM, https://omim.org/),^[18]^ and Therapeutic Target Database (http://db.idrblab.net/ttd/) for DKD-related genes. The results were integrated, and duplicated entries were removed.

### 2.5 Identification of “overlapping target genes”

The genes that were simultaneously targeted by rhubarb active compounds and implicated in DKD (hereafter referred to as overlapping target genes) were identified using Venny 2.1 (https://bioinfogp.cnb.csic.es/tools/venny/).^[19]^ Finally, a Venn diagram was drawn.

### 2.6 Construction of a “drug–target–disease” network diagram

We used the data on overlapping targets and interactions between the drugs, drug targets, and the disease to construct a “drug–target–disease” network using Cytoscape (version 3.7.2). Next, network topological parameters were obtained using the “network analyzer” function. A core architecture network was constructed using Cytoscape and the degree value of a node was represented as the number of edges connected to that node^[20]^—the most direct measurement of the centrality of a node. Betweenness centrality refers to the number of times a node serves as the shortest bridge between two other nodes; it is used to reveal the nodes with bridging functions in a network. A high betweenness centrality value of a node indicates its high tendency to serve as an “intermediary” and its significant role in a network.

### 2.7 Construction of a protein–protein interaction (PPI) network for target proteins and identification of core proteins

Next, a PPI network was constructed for overlapping target proteins using the STRING database (https://string-db.org/cgi/input?sessionId=bR2DXqok3ofm)^[21]^ and rhubarb–target interactions were identified. We restricted our research in the STRING database to “*Homo sapiens*” with a high confidence level of > 0.7. The results from Cytoscape analysis were calculated and the core proteins were identified using the degree value.

### 2.8 Gene ontology (GO) and Kyoto Encyclopedia of Genes and Genomes (KEGG) enrichment analyses of overlapping target proteins

We next performed GO and KEGG enrichment analyses of overlapping target proteins using the Database for Annotation, Visualization and Integrated Discovery Bioinformatics Resources 6.8 (https://david.ncifcrf.gov/).^[22–24]^ A *P* < .05 was used as the threshold, with a smaller value of *P* denoting significant enrichment of a GO or a KEGG term.

## 3 Results

### 3.1 Screening for rhubarb active compounds

We searched the TCMSP database using “rhubarb” as the keyword to obtain 92 related compounds. We filtered these compounds using the criteria OB > 30% and DL ≥ 0.18. In addition, we referred to the related literature to exclude the compounds without corresponding targets. Finally, a rhubarb active compound library containing 18 compounds was established. The basic information of these compounds is shown in Table 1.

**Table 1:** Basic information of 18 rhubarb active compounds identified in the study.

Molecule ID	Molecule name	OB (%)	DL
MOL000471	Aloe-emodin	83.38	0.24
MOL002299	DMR	68.62	0.02
MOL001456	Citric acid	56.22	0.05
MOL002235	Eupatin	50.80	0.41
MOL000096	(–)–Catechin	49.68	0.24
MOL002251	Mutatochrome	48.64	0.61
MOL002268	Rhein	47.07	0.28
MOL002281	Toralactone	46.46	0.24
MOL002288	Emodin-1-O-beta-D-glucopyranoside	44.81	0.8
MOL002301	DLA	44.51	0.01
MOL002259	Physciondiglucoside	41.65	0.63
MOL000358	Beta-sitosterol	36.91	0.75
MOL002303	Palmidin A	32.45	0.65
MOL002260	Procyanidin B-5,3’-O-gallate	31.99	0.32
MOL000346	Succinic acid	29.62	0.01
MOL000472	Emodin	24.40	0.24
MOL002295	Cinnamic acid	19.68	0.03
MOL002300	10beta-Hydroxy-6beta-isobutyrylfuranoeremophilane	16.94	0.29

### 3.2 Prediction of potential targets of rhubarb for treating DKD

The TCMSP database was used to extract 4,775 targets corresponding to active compounds of rhubarb; of these, the top 50 targets with the highest fix values were selected using PharmMapper. The nomenclatures of selected targets were standardized using UniProt. All data were integrated, and any duplicate entry was removed. Finally, 288 targets corresponding to active compounds of rhubarb were obtained.

**Figure 2:**
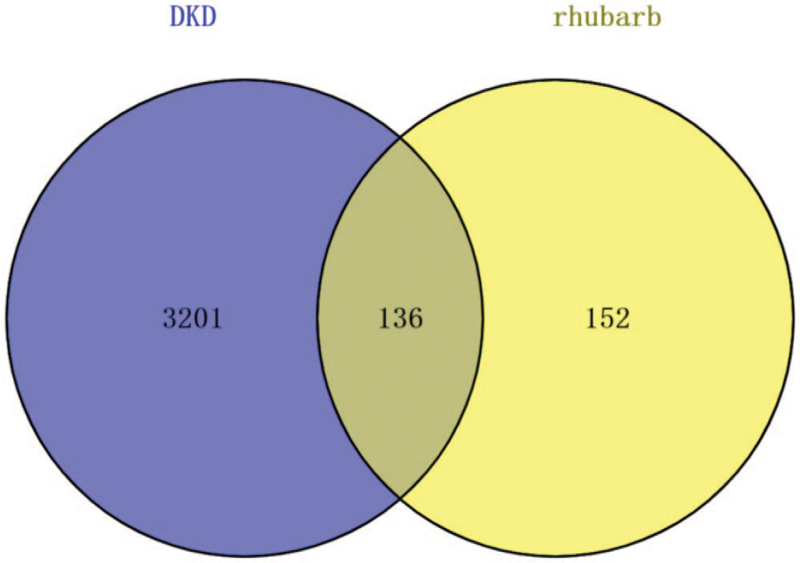
A total of 136 overlapping target genes were identified.

Using “DKD” as the keyword, 3320, 86, and 23 DKD-associated targets were obtained from GeneCards, OMIM, and Therapeutic Target Database databases, respectively. Next, 136 overlapping target genes were identified and visualized by Venny 2.1 (Fig. 2).

### 3.3 “Drug–target–disease” network diagram construction

**Figure 3:**
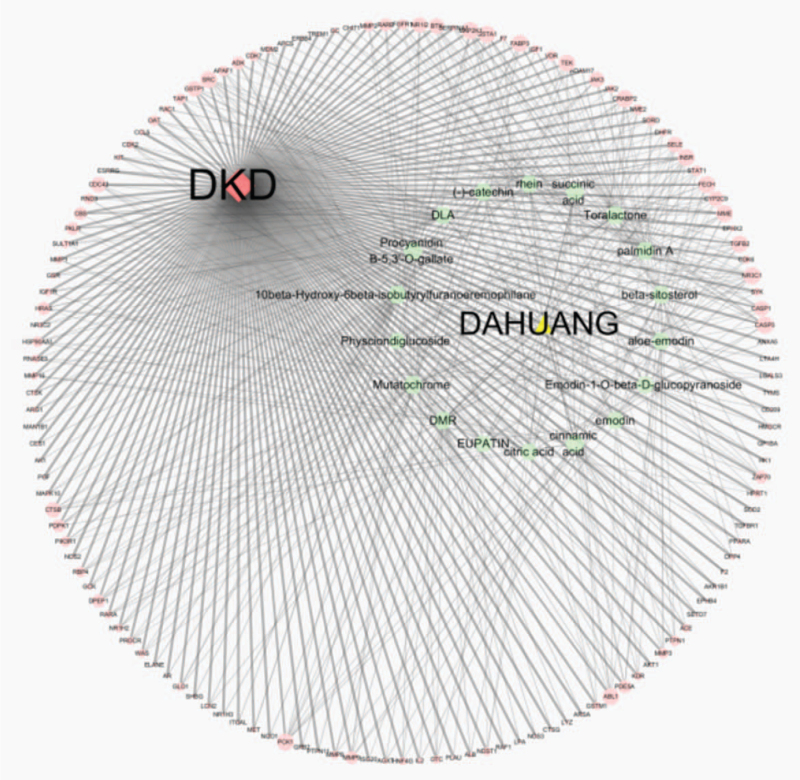
The “drug-target-disease” network diagram.

A “drug–target–disease” network diagram was constructed using Cytoscape (version 3.7.2) (Fig. 3). Several network topological parameters were obtained. As shown in Figure 3, the light green regular hexagons represent the active compounds of rhubarb, light pink circles represent the target genes of DKD, and lines between them represent the relationships between active compounds and DKD target genes. A more strong connection implied a closer relationship between the active compounds of rhubarb and DKD target genes.

The topological analysis revealed a network centralization value of 0.839, the network density value of 0.049, and the network heterogeneity value of 1.660. The shortest path was 24180 (100%). Based on the degree values and betweenness centrality of network nodes, the top five active compounds of rhubarb identified were mutatochrome (degree value = 32, betweenness centrality = 0.01914902), 10beta-hydroxy-6beta-isobutyrylfuranoeremophilane (degree value = 31, betweenness centrality = 0.01852226), toralactone (degree value = 31, betweenness centrality of 0.01880477), palmidin A (degree value = 29, betweenness centrality = 0.01503692), and cinnamic acid (degree value = 29, betweenness centrality = 0.01852707) (Table 2). The majority of active compounds of rhubarb were related to multiple target genes. For example, mutatochrome was related to 31 target genes, and 10beta-hydroxy-6beta-isobutyrylfuranoeremophilane was related to 30 target genes.

**Table 2:** Analysis of node degree and betweenness centrality values of active compounds of rhubarb.

Name	Betweenness centrality	Degree
Mutatochrome	0.01914902	32
10beta-Hydroxy-6beta-isobutyrylfuranoeremophilane	0.01852226	31
Toralactone	0.01880477	31
Palmidin A	0.01503692	29
Cinnamic acid	0.01852707	29
Beta-sitosterol	0.01450198	28
DMR	0.01722166	27
Procyanidin B-5,3’-O-gallate	0.01410908	26
(–)–Catechin	0.0123414	26
Aloe-emodin	0.01214795	25
Succinic acid	0.01325694	25
Rhein	0.01153864	24
DLA	0.01137659	23
Emodin-1-O-beta-D-glucopyranoside	0.01195699	23
Emodin	0.01070669	23
Citric acid	0.00900986	22
Eupatin	0.00848923	20
Physciondiglucoside	0.0038587	14

The top five target genes identified were *NR3C1* (degree value = 11, betweenness centrality = 0.00567111), *CASP3* (degree value = 10, betweenness centrality = 0.0048482), *INSR* (degree value = 10, betweenness centrality = 0.00581331), *CRABP2* (degree value = 10, betweenness centrality = 0.00424984), and *MAP2K1* (degree value = 10, betweenness centrality = 0.00510742). The majority of the target genes were associated with more than one compound. For example, *NR3C1* was associated with 10 compounds, and CASP3 was associated with 9 compounds.

### 3.4 Construction of a PPI network diagram for overlapping targets

**Figure 4:**
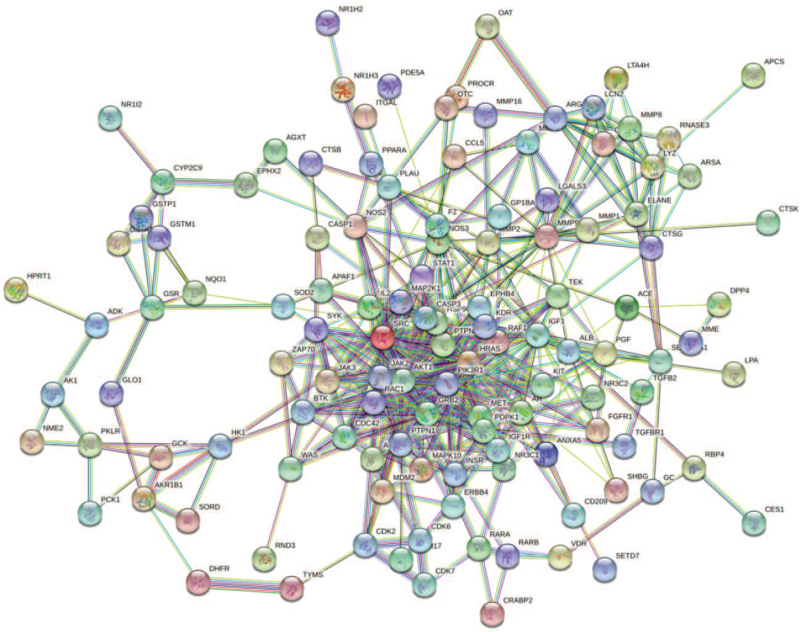
The PPI network diagram for the overlapping target proteins. PPI = protein-protein interaction.

**Figure 5:**
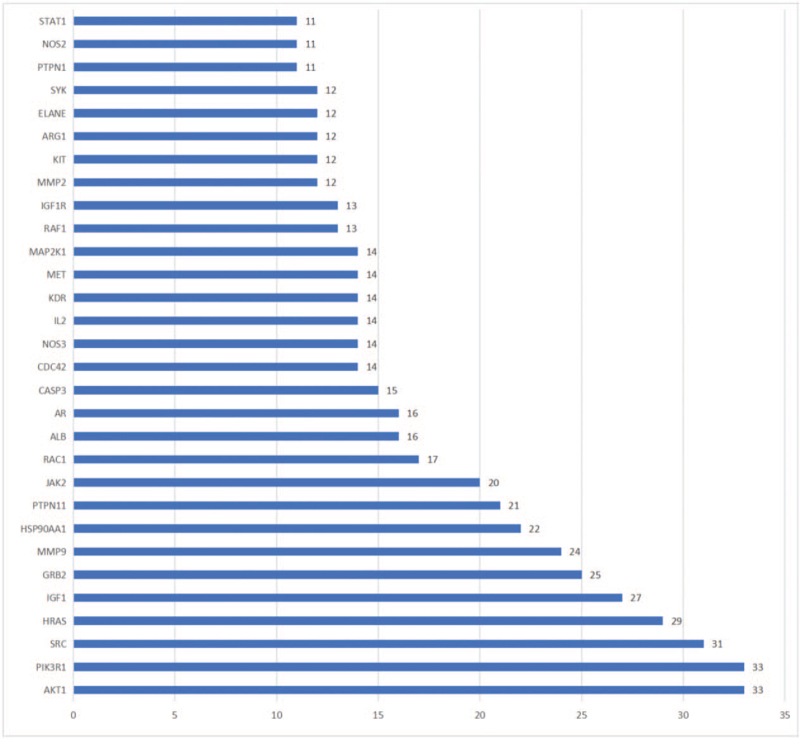
Degree values of the top 30 target proteins.

We used a high confidence level of > 0.7 and eliminated discrete targets to obtain a PPI network diagram for overlapping targets using the STRING database (Fig. 4). The PPI network included 136 nodes and 465 edges (average degree value of node = 6.84). The 30 most closely related target proteins are shown in Figure 5. Each node in the PPI network diagram represented a target protein, the connections between the nodes represented the interactions between target proteins, and different colors represented different types of interactions. A higher number of connections between the two target proteins indicated their close association. The PPI network analysis revealed a possible function of AKT1, PIK3R1, SRC, HRAS, IGF1, GRB2, and MMP9 in the molecular mechanism underlying the use of rhubarb in DKD treatment.

### 3.5 GO and KEGG pathway enrichment analyses of targets

**Figure 6:**
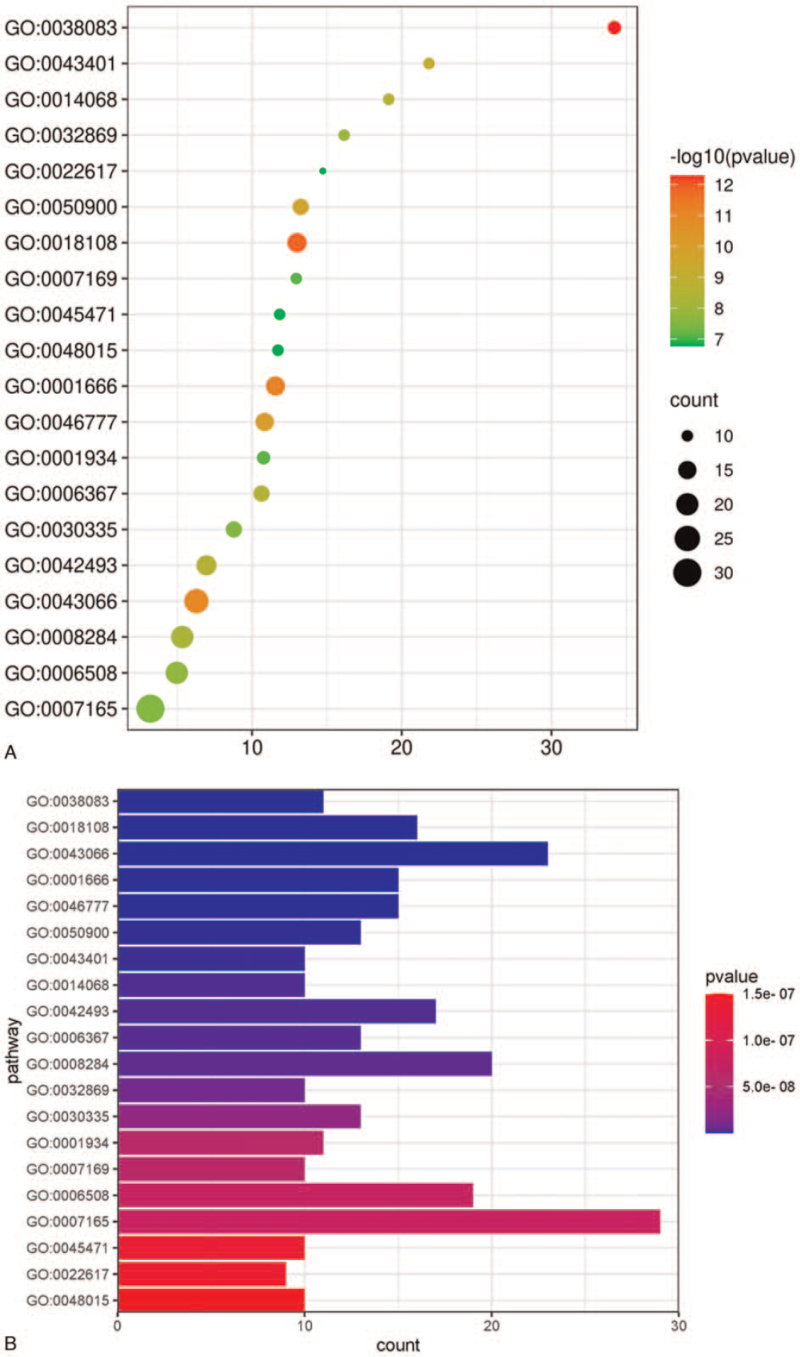
A. GO annotations of target genes of rhubarb implicated in DKD treatment. B. GO annotations of target genes of rhubarb implicated in DKD treatment. GO = gene ontology, DKD = diabetic kidney disease.

To identify the functions of overlapping targets, we performed GO and KEGG pathway enrichment analyses of overlapping targets. We obtained 352 GO annotated enriched terms by setting a threshold *P* value of < .05. The 20 GO annotation terms with the lowest P values are shown in Figure 6. The effects of rhubarb on DKD treatment could be related to autophosphorylation of peptidyl-tyrosine, phosphorylation of peptidyl-tyrosine, response to hypoxia, apoptosis inhibition, protein autophosphorylation, proteolysis, and steroid hormone-mediated signaling pathways.

**Figure 7:**
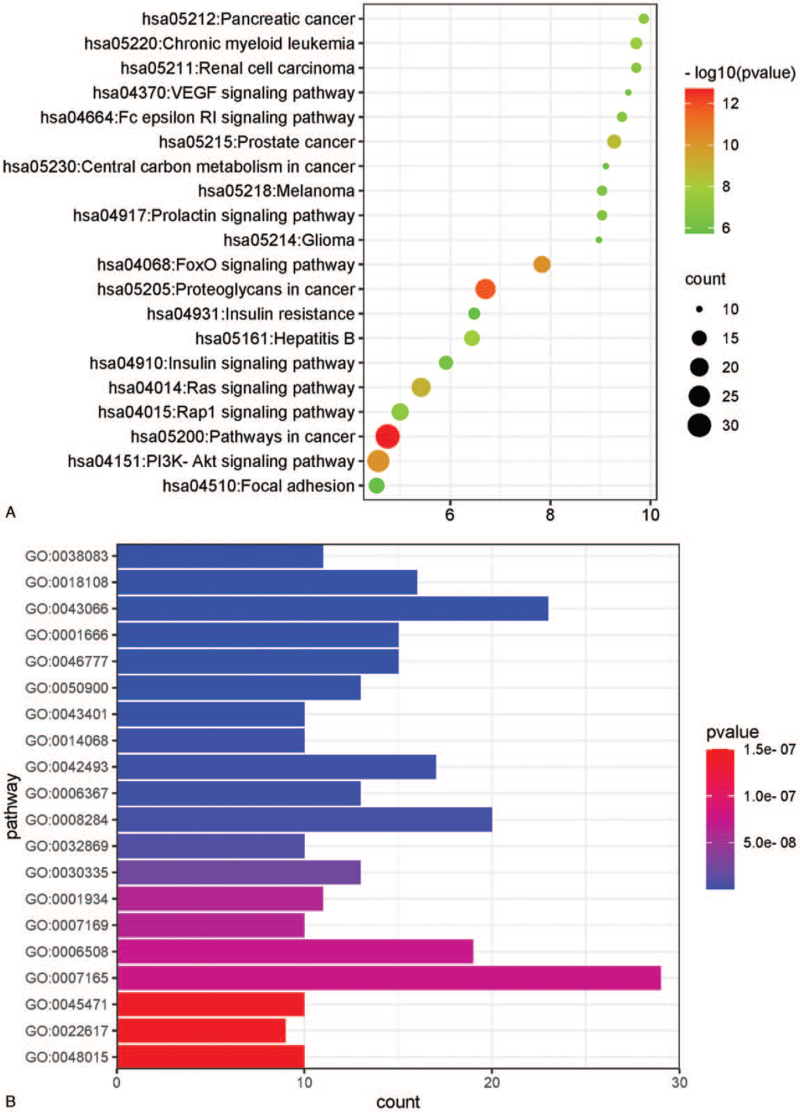
A. KEGG pathway enrichment analysis of target genes of rhubarb for treating DKD. B. KEGG pathway enrichment analysis of target genes of rhubarb for treating DKD. DKD = diabetic kidney disease, KEGG = Kyoto Encyclopedia of Genes and Genomes.

The KEGG pathway enrichment analysis resulted in 93 signaling pathways. The 20 signaling pathways with the lowest *P* values are shown in Figure 7. The highly enriched pathways included those involved in cancer, proteoglycans in cancer, the FOXO signaling pathway, the PI3K/Akt signaling pathway, and the Ras signaling pathway. The KEGG pathway enrichment analysis showed that except for the PI3K/Akt signaling pathway, the majority of the enriched pathways corresponded to cancer, inflammation, and viral infections. This finding indicated the potential of rhubarb in treating these disease conditions.

## 4 Discussion

Diabetic kidney disease, one of the microvascular complications associated with diabetes, is characterized by damage to small blood vessels. DKD can affect different kidney cells including glomerular epithelial cells, distal renal tubular cells, mesangial cells, and glomerular podocytes.^[25]^

Rhubarb, a herb, has been widely used in TCM. It is bitter in taste and cold in nature; raw rhubarb is known for its cooling effects. In addition, it detoxifies the blood, removes stagnation, increases blood circulation, and prevents blood stasis. Wine-processed rhubarb is used to detoxify the body and restore homeostasis. Recent studies have reported the use of major components and several prescriptions of rhubarb in the treatment of DKD.^[26–28]^

We extracted 18 rhubarb core active compounds and identified 136 target genes of rhubarb that could be used as potential therapeutic targets for DKD.

The primary active compounds of rhubarb included (–)–catechin, aloe-emodin, rhein, and emodin. Their mechanisms of intervention in treating DKD have previously been confirmed to a certain extent.^[29–32]^ For instance, Zhu et al^[33]^ reported that (–)–catechin blocked the inflammatory response pathways by scavenging methylglyoxal, thereby inhibiting the formation of advanced glycation end-products and improving the renal function in DKD mice. Similarly, Li et al^[34]^ stated that aloe-emodin effectively improved the renal function in DKD rats and exerted certain therapeutic and preventive effects on DKD. Another study demonstrated that rhein alleviated the autophagy of renal tubular cells in DKD mice by regulating the AMPK/mTOR pathway, thereby delaying the process of renal fibrosis.^[35]^ A study by Qi et al^[7]^ found that emodin reduced cell autophagy and alleviated oxidative damage in the kidneys of DKD mice by downregulating the expression of the mouse *miR-21* gene.

*CASP3* gene, which encodes Caspase-3, has been previously reported as a target gene of rhubarb in DKD treatment.^[36]^ Caspase-3 has been implicated in normal brain development. In addition, it is implicated in the execution stage of apoptosis, that is, chromosome condensation and DNA fragmentation.^[37]^ Apoptosis refers to autonomous programmed death of cells; abnormal cell apoptosis can lead to several diseases. For example, studies have reported the involvement of renal cell apoptosis in DKD.^[38–40]^ Caspase-3 can be activated through both extrinsic and intrinsic pathways of apoptosis.^[41–43]^ Both in vivo and in vitro studies have demonstrated that the inhibition of Caspase-3 activity by Z-DEVD-FMK effectively reduced the degree of renal tubular cell apoptosis.^[44]^ A previous study proved that Danggui Buxue decoction reduced the apoptosis of kidney cells in DKD rats by downregulating the expression of ATF6, CHOP, and Caspase-3.^[16]^

The results of our PPI network analysis revealed the involvement of AKT1, PIK3R1, SRC, HRAS, IGF1, GRB2, and MMP9 in the mechanism underlying the therapeutic effects of rhubarb in DKD treatment. The GO enrichment analysis resulted in 352 enriched GO annotations that corresponded to oxidation, cell apoptosis, and proteolysis, which were implicated in the treatment of DKD with rhubarb. Moreover, the involvement of multiple proteins and pathways reflects the diversity in the mechanism underlying the therapeutic effects of rhubarb in DKD treatment and provides a scientific rationale for future studies. The steroid hormone-mediated transduction is executed via several molecular signals generated by the binding between intracellular steroid hormone receptors and their physiological ligands. Proteolysis, a process of degrading proteins into shorter polypeptides or amino acids, maintains protein homeostasis. Therefore, abnormalities in proteolysis can cause diseases. For example, a previous study demonstrated that the abnormal activation of matriptase caused hydrolysis of podocin, thereby damaging podocytes.^[45]^ Steroid hormones can regulate inflammatory responses and immune and reproductive functions in the body. Natural steroid hormones are lipids that can easily pass through the cell membranes to bind to steroid hormone receptors, thereby causing cellular alterations. Insulin combined with steroid therapy can partially rescue diabetes-induced imbalance in hormones and angiogenesis.^[46]^

The KEGG pathway enrichment analysis revealed multiple signaling pathways to be implicated in the therapeutic effects exerted by rhubarb on DKD. The analysis indicated that rhubarb could be exerting its therapeutic effect on DKD by regulating the PI3K/Akt signaling pathway. Twenty-seven PI3K/Akt signaling pathway-related genes, namely *PIK3R1*, *IGF1R*, *KDR*, *AKT1*, *RAC1*, *JAK2*, *PCK1*, *HRAS*, *JAK3*, *MAP2K1*, *HSP90AA1*, *SYK*, *NOS3*, *PDPK1*, *INSR*, *IGF1*, *IL2*, *PGF*, *CDK6*, *KIT*, *CDK2*, *MDM2*, *GRB2*, *TEK*, *RAF1*, *MET*, and *FGFR1*, were found to be significantly enriched (*P* value = 7.12E-11). The PI3K/Akt signaling pathway transduces extracellular signals to promote cell metabolism, cell proliferation, cell growth, and angiogenesis. The major effector molecules involved in this pathway include receptor tyrosine kinases (RTKs), phosphatidylinositol-4,5-bisphosphate (PIP2), phosphatidylinositol 3-kinase (PI3K), AKT/protein kinase B, and phosphatidylinositol-3,4,5-bisphosphate. A study demonstrated that PAQR3 knockout inhibited the activation of the PI3K/Akt signaling pathway in glomerular mesangial cells following hyperglycemia, consequently inhibiting the deposition of extracellular matrix in glomerular mesangial cells.^[47]^ Animal experiments indicated that histone deacetylase (HDAC) inhibitors reduced the extracellular matrix deposition in the kidneys of diabetic mice and downregulated the expression of HDACs, TGF-β1, and α-SMA. In addition, METL14-mediated regulation of PTEN-induced activation of this pathway significantly affected the HDAC-induced epithelial–mesenchymal transition (EMT) of renal tubular cells in DKD.^[48]^ In addition, this pathway is related to the degree of renal fibrosis and glomerular podocyte damage.^[49–52]^

This study has the following limitations. First of all, network pharmacology research is based on databases and software. At present, the existing databases and software still have certain imperfections that may lead to imprecision of our conclusions, for example,the effect of different decoction time of Rhubarb on the active ingredients was not recorded in the database. Second, in experiments and clinical applications, different processing methods, decoction methods, and decoction time may cause changes in active ingredients and therapeutic targets of single Chinese medicines. These changes were not taken into consideration in our study and may thus cause imprecision of our conclusions. Third, our analysis of rhubarb active compounds was based on only TCMSP, which may result in selection bias. Fourth, this study only predicted the potential active ingredients, therapeutic targets and related signaling pathways of rhubarb in the treatment of DKD. The exact pharmacological effects of rhubarb on DKD need to be further validated through animal experiments and even clinical trials. Finally, the efficacy of drugs is based on medicinal substances, which must reach certain doses or concentrations to be effective. Therefore, cell or animal experiments are needed to test the efficacy of rhubarb in the treatment of DKD.

## 5 Conclusion

In this study, we adopted network pharmacological strategies to identify the active compounds and core target genes of rhubarb for treating DKD. A PPI network was constructed for the overlapping target proteins, followed by GO and KEGG pathway enrichment analyses of overlapping targets. Our results showed PI3K/Akt signaling pathway as the primary contributor to the mechanism underlying the therapeutic effect of rhubarb in treating DKD. In addition, we predicted other potential active components, core gene targets, and signaling pathways implicated in DKD treatment by rhubarb. Being a bioinformatic study, the active compounds, gene targets, and pathways identified and proposed in this study warrant further verification and validation by follow-up studies.

## Author contributions

**Conceptualization:** Zheng Nan.

**Data curation:** Yingyuan Gao.

**Formal analysis:** Yingyuan Gao.

**Funding acquisition:** Zheng Nan.

**Investigation:** Yingyuan Gao, Zheng Nan.

**Methodology:** Yingyuan Gao, Zheng Nan.

**Project administration:** Yingyuan Gao, Zheng Nan.

**Resources:** Yingyuan Gao, Zheng Nan.

**Software:** Yingyuan Gao.

**Supervision:** Zheng Nan.

**Validation:** Zheng Nan.

**Visualization:** Yingyuan Gao.

**Writing – original draft:** Yingyuan Gao.

**Writing – review & editing:** Zheng Nan.
